# Recent advances in circadian rhythms in cardiovascular system

**DOI:** 10.3389/fphar.2015.00071

**Published:** 2015-04-01

**Authors:** Lihong Chen, Guangrui Yang

**Affiliations:** ^1^The Institute for Translational Medicine and Therapeutics, Perelman School of Medicine, University of PennsylvaniaPhiladelphia, PA, USA; ^2^Department of Systems Pharmacology and Translational Therapeutics, Perelman School of Medicine, University of PennsylvaniaPhiladelphia, PA, USA

**Keywords:** circadian rhythm, circadian clock, CVDs, blood pressure, myocardial infarction

## Abstract

Growing evidence shows that intrinsic circadian clocks are tightly related to cardiovascular functions. The diurnal changes in blood pressure and heart rate are well known circadian rhythms. Endothelial function, platelet aggregation and thrombus formation exhibit circadian changes as well. The onset of many cardiovascular diseases (CVDs) or events, such as myocardial infarction, stroke, arrhythmia, and sudden cardiac death, also exhibits temporal trends. Furthermore, there is strong evidence from animal models and epidemiological studies showing that disruption of circadian rhythms is a significant risk factor for many CVDs, and the intervention of CVDs may have a time dependent effect. In this mini review, we summarized recent advances in our understanding of the relationship between circadian rhythm and cardiovascular physiology and diseases including blood pressure regulation and myocardial infarction.

## Introduction

**Figure 1:**
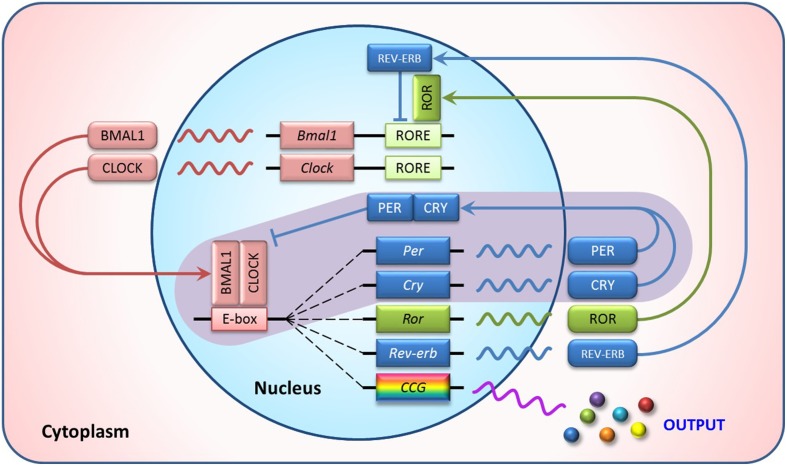
**Transcriptional feedback loops of the mammalian circadian clock**. In the core loop (purple background), BMAL1/CLOCK heterodimer activates transcription of the Per and Cry genes via binding to the E-box elements in their promoter regions. The resulting PER and CRY proteins heterodimerize, translocate to the nucleus and interact with the BMAL1/CLOCK complex to inhibit their own transcription. In addition, ROR activates and REV-ERB represses RORE-mediated transcription, forming the secondary autoregulatory feedback loops. This clock mechanism also controls rhythmic expression of numerous genes, called clock controlled genes (CCG), to perform biochemical or physiological roles in a circadian manner.

Circadian rhythms are biological processes displaying endogenous oscillations of about 24-h. These rhythms are widely observed in animals, plants, bacteria, and even cultured cells (Harmer et al., 2001). They are driven by a group of genes called clock genes. In mammals, the core clock genes consist of Bmal1 (Brain and muscle aryl-hydrocarbon receptor nuclear translocator-like 1), CLOCK (Circadian Locomotor Output Cycles Kaput), Per (Period), and Cry (Cryptochrome). They form a tightly regulated system with interlocking feedback and feed-forward loops (Figure 1) (Yang et al., 2013). BMAL1 and CLOCK proteins, or its paralog NPAS2 (neuronal PAS domain protein 2), form a heterodimer, bind to E-box elements in Per and Cry promoter regions and activate their transcription. Upon accumulation in the cytoplasm, PER and CRY proteins translocate to the nucleus where they repress the BMAL1:CLOCK/NPAS2 regulatory complex, thereby shutting down their own transcription. This core loop is interconnected with additional positive and negative regulatory loops involving nuclear receptors, such as RORα (RAR-related orphan receptor alpha), REV-ERBα (NR1D1, nuclear receptor subfamily 1, group D, member 1), and PPARs (Peroxisome proliferator-activated receptors). Additionally, these clock genes control numerous target genes (termed clock controlled genes, CCGs), thus regulating the circadian rhythms of various biochemical and physiological processes (Chen and Yang, 2014).

The circadian clock exists as the central clock in the suprachiasmatic nucleus (SCN) in the hypothalamus, and its peripheral tissues serve as the peripheral clock. The SCN receives light input from the retina, and then conveys the photic information into neural and/or humoral signals that orchestrate multifarious behavioral and biological rhythms, such as sleep-wake, hunger, body temperature, and hormone secretion cycles (Kohsaka et al., 2012). Although the SCN synchronizes internal time in various tissues, growing evidence from *in vitro* and *ex vivo* experiments has proved that the peripheral clock can function autonomously without central or systemic cues (Kowalska and Brown, 2007; Takeda and Maemura, 2011).

## Circadian clock in cardiovascular system

Circadian expression of clock genes in mouse heart (Young et al., 2001) and aorta (McNamara et al., 2001) were first described in 2001. Recently, Zhang et al. (2014) used a high temporal resolution of RNA-seq data and found that 6 and 4% of protein coding genes showed circadian rhythms in transcription in mouse heart and aorta, respectively. *Ex vivo* experiments displayed varied functions of mouse heart (Durgan et al., 2007) and aorta (Keskil et al., 1996; Prasai et al., 2013) that depended on the time the tissues were collected. In addition, human hearts were found to express clock genes in a time sensitive manner as well (Leibetseder et al., 2009). Furthermore, the observations of gene cycling were extended to cultured cells. In rat cardiomyocytes, the presence of 2.5% of fetal calf serum in culture medium is sufficient to maintain rhythmic expression of core clock genes Bmal1, Rev-erbα, and Per2 and energy metabolic genes pyruvate dehydrogenase kinase 4 and uncoupling protein 3 (Durgan et al., 2005). Functional clocks are also expressed in cultured endothelial cells (Takeda et al., 2007) and vascular smooth muscle cells (Nonaka et al., 2001). To study the role of circadian clocks in cardiovascular system, several tissue specific clock gene deletion mouse models were recently generated. For instance, cardiomyocyte deletion of Bmal1 results in abnormal electrocardiography with prolonged RR and QRS intervals (Schroder et al., 2013). The hearts from knockout mice were more susceptible to arrhythmia. Bmal1 deletion in endothelial cells (Westgate et al., 2008) or vascular smooth muscle cells (Xie et al., 2015) compromised the diurnal variation of blood pressure. These findings are consistent with the presence and importance of intrinsic clocks in cardiovascular system.

On the other hand, although all cell types in the cardiovascular system have intact molecular clocks, these peripheral clocks need to coordinate with the central clock to synchronize responsiveness of the heart and blood vessels to diurnal variations in their environment. The disruption of normal day-night cycles, such as jet lag, leads to desynchronization between central and peripheral clocks, heterogeneity of entrainment kinetics between different organs, and dysregulation of clock genes (Kiessling et al., 2010). Because circadian clocks control a large number of tissue specific CCGs (Zhang et al., 2014), the disruption of this mechanism will initiate a chain reaction to result in perturbation of a wide range of biochemical and physiological outputs, potentially contributing to the incidence of cardiovascular diseases (CVDs). For example, using a mouse model of pressure overload–induced cardiac hypertrophy, Martino et al. found that rhythm disturbance by housing mice under 10-h light: 10-h dark conditions adversely affected cardiac structure and function as well as altered expression of clock genes and cardiac remodeling genes (Martino et al., 2007). Interestingly and importantly, restoration of a normal 24-h diurnal rhythm could rescue these changes, suggesting that maintaining a normal rhythm is crucial to cardiovascular health.

## Circadian regulation of blood pressure

Day-night variations in blood pressure (BP) and heart rate (HR) are among the best known circadian rhythms of physiology. In humans, there is a 24-h variation in BP with a sharp rise before awakening, the highest BP value is around midmorning (Millar-Craig et al., 1978). Concomitantly, many cardiovascular events, such as sudden cardiac death, myocardial infarction and stroke, display diurnal variations with an increased incidence in the morning (Muller et al., 1985, 1987; Elliott, 1998; Reavey et al., 2013). These events, as well as kidney albuminuria and progression to end-stage renal diseases, are relatively common in patients whose blood pressure fails to decline during the night, so-called non-dippers (Takeda and Maemura, 2010). Inverse dippers—BP rises instead of decreases at night—showed even higher cardiovascular mortality (Kario et al., 2001). These time-dependent effects are not just consequences of the sleep/wakefulness cycle or the rhythms in neuroendocrine constituents, but are also believed to be attributed to the intrinsic properties of the hearts and blood vessels whose functions show significant fluctuations during the course of the day (Durgan and Young, 2010; Paschos and Fitzgerald, 2010).

**Figure 2:**
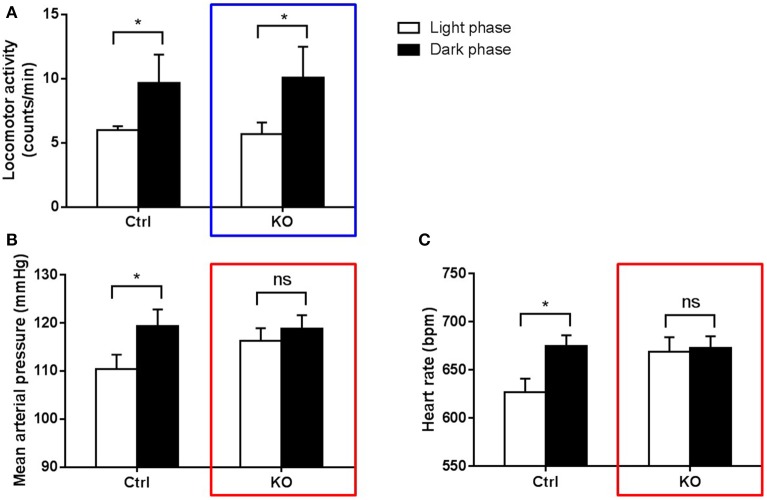
**Dissociation between behavior and BP regulation in circadian-disrupted mice (Yang et al., 2012)**. PPARγ knockout mice (KO) and their littermate controls (Ctrl) were kept under regular light/dark cycles. Locomotor activity **(A)**, mean arterial pressure **(B)** and heart rate **(C)** were recorded using radiotelemetry. Both KO (blue box) and control mice display obvious day/night variation in locomotor activity. However, KO mice cannot maintain normal variations in BP and heart rate (red boxes) as control mice. *p < 0.05; ns, not significant.

Studies in genetic manipulated mice have suggested the involvement of intrinsic circadian clock in BP rhythm regulation. One of the most interesting findings is the dissociation between behavior and BP regulation (Figure 2). As the closest phylogenetic neighbor of ROR and REV-ERB, nuclear receptor PPARγ regulates the circadian rhythms of BP and heart rate via direct interaction with Bmal1 gene (Wang et al., 2008b; Yang et al., 2012). Although both vascular and global PPARγ knockout mice responded to light well and displayed rhythmic behavior pattern under regular light/dark conditions, the diurnal variations of BP was dampened or even abolished in these knockout mice. This striking dissociation between physiology and behavior strongly suggests that intrinsic clocks inside the blood vessels contribute to their functions that fluctuate in a 24-h cycle. Several other core clock genes were also reported to regulate BP in various ways. Global deletion of Bmal1 in mice abolishes the circadian rhythm of BP, which is accompanied by hypotension likely due to the reduced production of catecholamines (Curtis et al., 2007) or the lack of Bmal1 in vascular smooth muscle cells (Xie et al., 2015). By contrast, double deletion of Cry1/2 genes in mice give rise to salt-sensitive hypertension (Masuki et al., 2005).

It is worthwhile to note that the intrinsic circadian regulation of BP in humans remains to be determined. Kerkhof et al. failed to detect a significant 24-h variation of blood pressure in human when individuals were subjected to a 26-h constant light condition, while the heart rate exhibited a significant circadian pattern (Kerkhof et al., 1998). On the contrary, Scheer et al. found that, independent of environmental and behavioral changes, the endogenous circadian system modulates diurnal BP variation in humans (Scheer et al., 2010), while circadian misalignment by scheduling a recurring 28-h “day” for 8 days will induce hypertension and other adverse cardio-metabolic implications (Scheer et al., 2009).

Conventionally, most of the hypertensive patients were treated with anti-hypertensive medications in the mornings. Based on the consideration of the day-night variation of BP and the previous studies showing BP lowering effect of low dose of aspirin at bedtime (Hermida et al., 2003, 2009), Hermida et al., compared the potential differential reduction of CVD morbidity and mortality risk by a bedtime vs. upon-awakening hypertension treatment schedule in a large-scale (2156 untreated hypertensive subjects) and long-term (median follow-up of 5.6 years) study. They found that the patients who took at least one regular antihypertensive medication at bedtime gained better BP control and exhibited a significant reduction in CVD risk (Hermida et al., 2010). Although their results were impressive, independent antihypertension studies and intensive studies on hypertension-related complications are required to confirm the time-dependent effects of antihypertensive drugs and to establish chronotherapy to manage in hypertension.

## Circadian rhythms and myocardial infarction

Circadian rhythms in timing of onset and tolerance to myocardial infarction (MI) have been well established. It has been reported that the occurrence of MI is two to three times more frequent in the morning than at night (Muller et al., 1985; Culic, 2014). In the early morning, the increased systolic BP and HR results in an increased energy and oxygen demand by the heart, while the vascular tone of the coronary artery rises in the morning, resulting in a decreased coronary blood flow and oxygen supply. This mismatch between supply and demand elicits the high frequency of the onset of MI. In addition, plasminogen activator inhibitor-1 (Kurnik, 1995) and many platelet surface activation markers such as GPIb and P-selectin (Scheer et al., 2011) displayed a circadian pattern with high levels in the morning, which is coincident with the morning peak of thrombus formation and platelet aggregation (Tofler et al., 1987; Scheer and Shea, 2014). The resulting hypercoagulability partially underlies the morning onset of MI. Disruption of circadian rhythm like shiftwork and jetlag has been well established to be a risk factor for many CVDs, including MI (Knutsson et al., 1999). Even a 1 h shift, such as the transition from regular time to daylight saving time, can significantly increase the chances of MI occurring (Janszky and Ljung, 2008).

A series of cardiac functions related to the heart remodeling after MI are also known to have circadian variation. The early healing after MI relies on coordinated removal of necrotic tissues through an early inflammatory phase (Frangogiannis, 2012), followed by replacement and remodeling of the myocardium and extracellular matrix deposition (Liehn et al., 2011). As remodeling progresses toward the maturation phase, the heart undergoes size, shape and structure changes, which lead to ventricular dilation, dysfunction, and ultimately failure (Liehn et al., 2011). Most recently, Alibhai et al. (2014) demonstrated that short-term disruption of diurnal rhythms after myocardial infarction adversely affected the early inflammatory phase of left ventricular remodeling, altered the innate immune infiltration and scar formation, and eventually led to exacerbated maladaptive cardiac remodeling in mice. In contrast, maintaining normal rhythms throughout the course of the disease better preserved cardiac structure and function. Although no animal model can completely reflect patient experience, maintenance of normal diurnal rhythm during the recovery phase after MI should still aid in a coordinated and effective infarct healing response and improve patient outcome.

Moreover, clock genes may also exert non-clock roles in the cardiovascular system, which should be taken into account when interpreting the effect of circadian disruption. For instance, activation of an adenosine receptor Adora2b acts via Per2, but not other clock genes, to induce an energy utilization switch from fatty acid to glucose in cardiomyocytes, which promotes glycolysis and protects against cardiac ischemic injury (Eckle et al., 2012; Yang and Fitzgerald, 2012).

## Circadian rhythms and other cardiovascular diseases

Numerous animal models and human epidemiological studies also proved the adverse effects of circadian disruption in other CVDs. Mouse hearts in rhythm-disruptive environments are prone to malfunctions with altered clock gene cycling and reduced contractility (Martino et al., 2007). Clock gene deletion or mutation in mice dampened cardiovascular circadian rhythms accompanied by dilated cardiomyopathy (Lefta et al., 2012), arterial stiffness (Anea et al., 2010), or endothelial dysfunction (Viswambharan et al., 2007; Wang et al., 2008a; Anea et al., 2009). Impaired cholesterol metabolism and increased development of atherosclerosis was also verified in CLOCK mutant mice on a western as well as a normal diet (Pan et al., 2013). Aortic grafts from Bmal1 knockout mice transplanted into wild type mice developed robust arteriosclerosis without affecting systemic hemodynamics (Cheng et al., 2011). This data suggests that the intrinsic circadian clocks in blood vessels exert significant roles as an autonomous influence in arteriosclerotic diseases.

On the other hand, CVDs affect clock gene expression as well. For example, in salt sensitive rats, high salt diet induced cardiac hypertrophy is associated with attenuated rhythmic expression of core clock genes (Mohri et al., 2003). Aortic constriction induced pressure overload, which decreased the amplitude of circadian expression of clock genes in the rat heart (Young et al., 2001; Durgan et al., 2005). In a type 2 diabetic rat model, cardiac clock genes exhibited a phase shift with a 3 h delay, suggesting a loss of normal synchronization in diabetic hearts (Young et al., 2002). However, in high fat diet induced obese mice, vascular tissues are less sensitive to pathological disruption of circadian clocks than adipose tissue (Prasai et al., 2013). This evidence raises the possibility that although all cardiovascular cell types possess functional circadian clocks, this mechanism may be regulated in a cell-type specific manner. Desynchronization between different organs (e.g., heart and aorta) or cell types (e.g., VSMCs and ECs) could occur during specific physiological/pathological situations and may give an increased chance of CVDs.

Moreover, the day-night variations of blood pressure, heart rate and baroreflex sensitivity (a homeostatic mechanism for maintaining blood pressure) also coincide with diurnal variability in many other CVDs or events, such as cardiac arrhythmias, atherosclerosis and sudden death (Portaluppi et al., 2012; Yang et al., 2013). The timing of sudden cardiac death displayed circadian variability. It has a circadian pattern prominent in the early morning similar to that described in patients with coronary artery disease (Muller et al., 1987). Both atrial and ventricular arrhythmias appear to exhibit circadian patterning as well, with a higher frequency during the day than at night (Portaluppi et al., 2012). In hospital, many arrhythmias are observed as a consequence of MI. More complicatedly, circadian disruption not only impairs cardiovascular functions, but has also been linked to other diseases such as obesity, diabetes, immune disorders, mental illness that may affect each other (Harrington, 2010). Therefore, controlling or prevent the diseases that are related both to circadian rhythm and to cardiovascular functions becomes very important.

## Conclusion

Cardiovascular disease is the leading cause of death in many industrialized countries. Intensive effort has been made to understand the basic mechanisms. One field of investigation in recent years is the study of circadian rhythms. Increasing evidence has shown adverse effects of circadian disruption in the cardiovascular system. It becomes more and more evident and important in the modern age, particularly in developed countries, due to frequent disruptions to normal rhythms caused by shift work, artificial light, transmeridian air flight, and social activities (Boggild and Knutsson, 1999; Knutsson and Boggild, 2000).

The circadian rhythms not only affect health, but also drug efficiency. It's not surprising that some drugs for treating CVDs have been reported to exhibit time dependent effects since there's eminent circadian function of heart and blood vessels driven by both systemic and intrinsic clocks. Although several other mechanisms outside the cardiovascular system, such as chronopharmacokinetics (Musiek and Fitzgerald, 2013), have been suggested, the circadian clock within the heart and blood vessels should not be overlooked. Time dependent effects should be investigated when developing new drugs for CVDs.

### Conflict of interest statement

The authors declare that the research was conducted in the absence of any commercial or financial relationships that could be construed as a potential conflict of interest.

## References

[B1] Alibhai F. J. Tsimakouridze E. V. Chinnappareddy N. Wright D. C. Billia F. O'Sullivan M. L.. (2014). Short-term disruption of diurnal rhythms after murine myocardial infarction adversely affects long-term myocardial structure and function. Circ. Res. 114, 1713–1722. 10.1161/CIRCRESAHA.114.302995 24687134

[B2] Anea C. B. Ali M. I. Osmond J. M. Sullivan J. C. Stepp D. W. Merloiu A. M.. (2010). Matrix metalloproteinase 2 and 9 dysfunction underlie vascular stiffness in circadian clock mutant mice. Arterioscler. Thromb. Vasc. Biol. 30, 2535–2543. 10.1161/ATVBAHA.110.214379 20829506 PMC2988111

[B3] Anea C. B. Zhang M. Stepp D. W. Simkins G. B. Reed G. Fulton D. J.. (2009). Vascular disease in mice with a dysfunctional circadian clock. Circulation 119, 1510–1517. 10.1161/CIRCULATIONAHA.108.827477 19273720 PMC2761686

[B4] Boggild H. Knutsson A. (1999). Shift work, risk factors and cardiovascular disease. Scand. J. Work Environ. Health 25, 85–99. 10.5271/sjweh.410 10360463

[B5] Chen L. Yang G. (2014). PPARs integrate the mammalian clock and energy metabolism. PPAR Res. 2014:653017. 10.1155/2014/653017 24693278 PMC3945976

[B6] Cheng B. Anea C. B. Yao L. Chen F. Patel V. Merloiu A.. (2011). Tissue-intrinsic dysfunction of circadian clock confers transplant arteriosclerosis. Proc. Natl. Acad. Sci. U.S.A. 108, 17147–17152. 10.1073/pnas.1112998108 21969583 PMC3193243

[B7] Culic V. (2014). Chronobiological rhythms of acute cardiovascular events and underlying mechanisms. Int. J. Cardiol. 174, 417–419. 10.1016/j.ijcard.2014.04.043 24768379

[B8] Curtis A. M. Cheng Y. Kapoor S. Reilly D. Price T. S. Fitzgerald G. A. (2007). Circadian variation of blood pressure and the vascular response to asynchronous stress. Proc. Natl. Acad. Sci. U.S.A. 104, 3450–3455. 10.1073/pnas.0611680104 17360665 PMC1802007

[B9] Durgan D. J. Hotze M. A. Tomlin T. M. Egbejimi O. Graveleau C. Abel E. D.. (2005). The intrinsic circadian clock within the cardiomyocyte. Am. J. Physiolog. 289, H1530–H1541. 10.1152/ajpheart.00406.2005 15937094

[B10] Durgan D. J. Moore M. W. Ha N. P. Egbejimi O. Fields A. Mbawuike U.. (2007). Circadian rhythms in myocardial metabolism and contractile function: influence of workload and oleate. Am. J. Physiol. Heart Circ. Physiol. 293, H2385–H2393. 10.1152/ajpheart.01361.2006 17616739

[B11] Durgan D. J. Young M. E. (2010). The cardiomyocyte circadian clock: emerging roles in health and disease. Circ. Res. 106, 647–658. 10.1161/CIRCRESAHA.109.209957 20203314 PMC3223121

[B12] Eckle T. Hartmann K. Bonney S. Reithel S. Mittelbronn M. Walker L. A.. (2012). Adora2b-elicited Per2 stabilization promotes a HIF-dependent metabolic switch crucial for myocardial adaptation to ischemia. Nat. Med. 18, 774–782. 10.1038/nm.2728 22504483 PMC3378044

[B13] Elliott W. J. (1998). Circadian variation in the timing of stroke onset: a meta-analysis. Stroke 29, 992–996. 10.1161/01.STR.29.5.992 9596248

[B14] Frangogiannis N. G. (2012). Regulation of the inflammatory response in cardiac repair. Circ. Res. 110, 159–173. 10.1161/CIRCRESAHA.111.243162 22223212 PMC3690135

[B15] Harmer S. L. Panda S. Kay S. A. (2001). Molecular bases of circadian rhythms. Annu. Rev. Cell Dev. Biol. 17, 215–253. 10.1146/annurev.cellbio.17.1.215 11687489

[B16] Harrington M. (2010). Location, location, location: important for jet-lagged circadian loops. J. Clin. Invest. 120, 2265–2267. 10.1172/JCI43632 20577055 PMC2898616

[B17] Hermida R. C. Ayala D. E. Calvo C. Lopez J. E. Fernandez J. R. Mojon A.. (2003). Administration time-dependent effects of aspirin on blood pressure in untreated hypertensive patients. Hypertension 41, 1259–1267. 10.1161/01.HYP.0000072335.73748.0D 12732586

[B18] Hermida R. C. Ayala D. E. Mojon A. Fernandez J. R. (2009). Ambulatory blood pressure control with bedtime aspirin administration in subjects with prehypertension. Am. J. Hypertens. 22, 896–903. 10.1038/ajh.2009.83 19407805

[B19] Hermida R. C. Ayala D. E. Mojon A. Fernandez J. R. (2010). Influence of circadian time of hypertension treatment on cardiovascular risk: results of the MAPEC study. Chronobiol. Int. 27, 1629–1651. 10.3109/07420528.2010.510230 20854139

[B20] Janszky I. Ljung R. (2008). Shifts to and from daylight saving time and incidence of myocardial infarction. N. Engl. J. Med. 359, 1966–1968. 10.1056/NEJMc0807104 18971502

[B21] Kario K. Pickering T. G. Matsuo T. Hoshide S. Schwartz J. E. Shimada K. (2001). Stroke prognosis and abnormal nocturnal blood pressure falls in older hypertensives. Hypertension 38, 852–857. 10.1161/hy1001.092640 11641298

[B22] Kerkhof G. A. Van Dongen H. P. Bobbert A. C. (1998). Absence of endogenous circadian rhythmicity in blood pressure? Am. J. Hypertens. 11, 373–377. 10.1016/S0895-7061(97)00461-5 9544879

[B23] Keskil Z. Gorgun C. Z. Hodoglugil U. Zengil H. (1996). Twenty-four-hour variations in the sensitivity of rat aorta to vasoactive agents. Chronobiol. Int. 13, 465–475. 10.3109/07420529609020917 8974192

[B24] Kiessling S. Eichele G. Oster H. (2010). Adrenal glucocorticoids have a key role in circadian resynchronization in a mouse model of jet lag. J. Clin. Invest. 120, 2600–2609. 10.1172/JCI41192 20577050 PMC2898589

[B25] Knutsson A. Boggild H. (2000). Shiftwork and cardiovascular disease: review of disease mechanisms. Rev. Environ. Health 15, 359–372. 10.1515/REVEH.2000.15.4.359 11199246

[B26] Knutsson A. Hallquist J. Reuterwall C. Theorell T. Akerstedt T. (1999). Shiftwork and myocardial infarction: a case-control study. Occup. Environ. Med. 56, 46–50. 10.1136/oem.56.1.46 10341746 PMC1757657

[B27] Kohsaka A. Waki H. Cui H. Gouraud S. S. Maeda M. (2012). Integration of metabolic and cardiovascular diurnal rhythms by circadian clock. Endocr. J. 59, 447–456. 10.1507/endocrj.EJ12-0057 22361995

[B28] Kowalska E. Brown S. A. (2007). Peripheral clocks: keeping up with the master clock. Cold Spring Harb. Symp. Quant. Biol. 72, 301–305. 10.1101/sqb.2007.72.014 18419287

[B29] Kurnik P. B. (1995). Circadian variation in the efficacy of tissue-type plasminogen activator. Circulation 91, 1341–1346. 10.1161/01.CIR.91.5.1341 7867171

[B30] Lefta M. Campbell K. S. Feng H. Z. Jin J. P. Esser K. A. (2012). Development of dilated cardiomyopathy in Bmal1-deficient mice. Am. J. Physiol. Heart Circ. Physiol. 303, H475–H485. 10.1152/ajpheart.00238.2012 22707558 PMC3423146

[B31] Leibetseder V. Humpeler S. Svoboda M. Schmid D. Thalhammer T. Zuckermann A.. (2009). Clock genes display rhythmic expression in human hearts. Chronobiol. Int. 26, 621–636. 10.1080/07420520902924939 19444745

[B32] Liehn E. A. Postea O. Curaj A. Marx N. (2011). Repair after myocardial infarction, between fantasy and reality: the role of chemokines. J. Am. Coll. Cardiol. 58, 2357–2362. 10.1016/j.jacc.2011.08.034 22115639

[B33] Martino T. A. Tata N. Belsham D. D. Chalmers J. Straume M. Lee P.. (2007). Disturbed diurnal rhythm alters gene expression and exacerbates cardiovascular disease with rescue by resynchronization. Hypertension 49, 1104–1113. 10.1161/HYPERTENSIONAHA.106.083568 17339537

[B34] Masuki S. Todo T. Nakano Y. Okamura H. Nose H. (2005). Reduced alpha-adrenoceptor responsiveness and enhanced baroreflex sensitivity in Cry-deficient mice lacking a biological clock. J. Physiol. 566, 213–224. 10.1113/jphysiol.2005.086728 15860530 PMC1464725

[B35] McNamara P. Seo S. B. Rudic R. D. Sehgal A. Chakravarti D. FitzGerald G. A. (2001). Regulation of CLOCK and MOP4 by nuclear hormone receptors in the vasculature: a humoral mechanism to reset a peripheral clock. Cell 105, 877–889. 10.1016/S0092-8674(01)00401-9 11439184

[B36] Millar-Craig M. W. Bishop C. N. Raftery E. B. (1978). Circadian variation of blood-pressure. Lancet 1, 795–797. 10.1016/S0140-6736(78)92998-7 85815

[B37] Mohri T. Emoto N. Nonaka H. Fukuya H. Yagita K. Okamura H.. (2003). Alterations of circadian expressions of clock genes in Dahl salt-sensitive rats fed a high-salt diet. Hypertension 42, 189–194. 10.1161/01.HYP.0000082766.63952.49 12835331

[B38] Muller J. E. Ludmer P. L. Willich S. N. Tofler G. H. Aylmer G. Klangos I.. (1987). Circadian variation in the frequency of sudden cardiac death. Circulation 75, 131–138. 10.1161/01.CIR.75.1.131 3791599

[B39] Muller J. E. Stone P. H. Turi Z. G. Rutherford J. D. Czeisler C. A. Parker C.. (1985). Circadian variation in the frequency of onset of acute myocardial infarction. N. Engl. J. Med. 313, 1315–1322. 10.1056/NEJM198511213132103 2865677

[B40] Musiek E. S. Fitzgerald G. A. (2013). Molecular clocks in pharmacology. Handb. Exp. Pharmacol. 217, 243–260. 10.1007/978-3-642-25950-0_10 23604482 PMC3684693

[B41] Nonaka H. Emoto N. Ikeda K. Fukuya H. Rohman M. S. Raharjo S. B.. (2001). Angiotensin II induces circadian gene expression of clock genes in cultured vascular smooth muscle cells. Circulation 104, 1746–1748. 10.1161/hc4001.098048 11591607

[B42] Pan X. Jiang X. C. Hussain M. M. (2013). Impaired cholesterol metabolism and enhanced atherosclerosis in clock mutant mice. Circulation 128, 1758–1769. 10.1161/CIRCULATIONAHA.113.002885 24014832 PMC3897228

[B43] Paschos G. K. Fitzgerald G. A. (2010). Circadian clocks and vascular function. Circ. Res. 106, 833–841. 10.1161/CIRCRESAHA.109.211706 20299673 PMC2848505

[B44] Portaluppi F. Tiseo R. Smolensky M. H. Hermida R. C. Ayala D. E. Fabbian F. (2012). Circadian rhythms and cardiovascular health. Sleep Med. Rev. 16, 151–166. 10.1016/j.smrv.2011.04.003 21641838

[B45] Prasai M. J. Mughal R. S. Wheatcroft S. B. Kearney M. T. Grant P. J. Scott E. M. (2013). Diurnal variation in vascular and metabolic function in diet-induced obesity: divergence of insulin resistance and loss of clock rhythm. Diabetes 62, 1981–1989. 10.2337/db11-1740 23382450 PMC3661613

[B46] Reavey M. Saner H. Paccaud F. Marques-Vidal P. (2013). Exploring the periodicity of cardiovascular events in Switzerland: variation in deaths and hospitalizations across seasons, day of the week and hour of the day. Int. J. Cardiol. 168, 2195–2200. 10.1016/j.ijcard.2013.01.224 23452890

[B47] Scheer F. A. Hilton M. F. Mantzoros C. S. Shea S. A. (2009). Adverse metabolic and cardiovascular consequences of circadian misalignment. Proc. Natl. Acad. Sci. U.S.A. 106, 4453–4458. 10.1073/pnas.0808180106 19255424 PMC2657421

[B48] Scheer F. A. Hu K. Evoniuk H. Kelly E. E. Malhotra A. Hilton M. F.. (2010). Impact of the human circadian system, exercise, and their interaction on cardiovascular function. Proc. Natl. Acad. Sci. U.S.A. 107, 20541–20546. 10.1073/pnas.1006749107 21059915 PMC2996667

[B49] Scheer F. A. Michelson A. D. Frelinger A. L. III. Evoniuk H. Kelly E. E. McCarthy M.. (2011). The human endogenous circadian system causes greatest platelet activation during the biological morning independent of behaviors. PLoS ONE 6:e24549. 10.1371/journal.pone.0024549 21931750 PMC3169622

[B50] Scheer F. A. Shea S. A. (2014). Human circadian system causes a morning peak in prothrombotic plasminogen activator inhibitor-1 (PAI-1) independent of the sleep/wake cycle. Blood 123, 590–593. 10.1182/blood-2013-07-517060 24200683 PMC3901072

[B51] Schroder E. A. Lefta M. Zhang X. Bartos D. C. Feng H. Z. Zhao Y. (2013). The cardiomyocyte molecular clock, regulation of Scn5a, and arrhythmia susceptibility. American journal of physiology. Cell Physiol. 304, C954–C965 10.1152/ajpcell.00383.2012 PMC3651636 23364267

[B52] Takeda N. Maemura K. (2010). Circadian clock and vascular disease. Hypert. Res. 33, 645–651. 10.1038/hr.2010.68 20448639

[B53] Takeda N. Maemura K. (2011). Circadian clock and cardiovascular disease. J. Cardiol. 57, 249–256. 10.1016/j.jjcc.2011.02.006 21441015

[B54] Takeda N. Maemura K. Horie S. Oishi K. Imai Y. Harada T.. (2007). Thrombomodulin is a clock-controlled gene in vascular endothelial cells. J. Biol. Chem. 282, 32561–32567. 10.1074/jbc.M705692200 17848551

[B55] Tofler G. H. Brezinski D. Schafer A. I. Czeisler C. A. Rutherford J. D. Willich S. N.. (1987). Concurrent morning increase in platelet aggregability and the risk of myocardial infarction and sudden cardiac death. N. Engl. J. Med. 316, 1514–1518. 10.1056/NEJM198706113162405 3587281

[B56] Viswambharan H. Carvas J. M. Antic V. Marecic A. Jud C. Zaugg C. E.. (2007). Mutation of the circadian clock gene Per2 alters vascular endothelial function. Circulation 115, 2188–2195. 10.1161/CIRCULATIONAHA.106.653303 17404161

[B57] Wang C. Y. Wen M. S. Wang H. W. Hsieh I. C. Li Y. Liu P. Y.. (2008a). Increased vascular senescence and impaired endothelial progenitor cell function mediated by mutation of circadian gene Per2. Circulation 118, 2166–2173. 10.1161/CIRCULATIONAHA.108.790469 18981300 PMC2656770

[B58] Wang N. Yang G. Jia Z. Zhang H. Aoyagi T. Soodvilai S.. (2008b). Vascular PPARgamma controls circadian variation in blood pressure and heart rate through Bmal1. Cell Metab. 8, 482–491. 10.1016/j.cmet.2008.10.009 19041764 PMC5484540

[B59] Westgate E. J. Cheng Y. Reilly D. F. Price T. S. Walisser J. A. Bradfield C. A.. (2008). Genetic components of the circadian clock regulate thrombogenesis *in vivo*. Circulation 117, 2087–2095. 10.1161/CIRCULATIONAHA.107.739227 18413500

[B60] Xie Z. Su W. Liu S. Zhao G. Esser K. Schroder E. A.. (2015). Smooth-muscle BMAL1 participates in blood pressure circadian rhythm regulation. J. Clin. Invest. 125, 324–336. 10.1172/JCI76881 25485682 PMC4382248

[B61] Yang G. Fitzgerald G. A. (2012). (Almost) Everything is illuminated: adenosine shines a light on cardioprotection. Circ. Res. 111, 965–966. 10.1161/CIRCRESAHA.112.279752 23023508

[B62] Yang G. Jia Z. Aoyagi T. McClain D. Mortensen R. M. Yang T. (2012). Systemic PPARgamma deletion impairs circadian rhythms of behavior and metabolism. PLoS ONE 7:e38117. 10.1371/journal.pone.0038117 22899986 PMC3416825

[B63] Yang G. Paschos G. Curtis A. M. Musiek E. S. McLoughlin S. C. Fitzgerald G. A. (2013). Knitting up the raveled sleave of care. Sci. Transl. Med. 5, 212rv213. 10.1126/scitranslmed.3007225 24259052

[B64] Young M. E. Razeghi P. Taegtmeyer H. (2001). Clock genes in the heart: characterization and attenuation with hypertrophy. Circ. Res. 88, 1142–1150. 10.1161/hh1101.091190 11397780

[B65] Young M. E. Wilson C. R. Razeghi P. Guthrie P. H. Taegtmeyer H. (2002). Alterations of the circadian clock in the heart by streptozotocin-induced diabetes. J. Mol. Cell. Cardiol. 34, 223–231. 10.1006/jmcc.2001.1504 11851361

[B66] Zhang R. Lahens N. F. Ballance H. I. Hughes M. E. Hogenesch J. B. (2014). A circadian gene expression atlas in mammals: implications for biology and medicine. Proc. Natl. Acad. Sci. U.S.A. 111, 16219–16224. 10.1073/pnas.1408886111 25349387 PMC4234565

